# Geographic Visualization of Mortality in the United States as Related to Healthcare Access by County

**DOI:** 10.7759/cureus.12820

**Published:** 2021-01-20

**Authors:** Jason Widrich, Shelley Nation, Prithvi Chippada, Eric Wiener, Eldon Jenkins, Landan Peters

**Affiliations:** 1 Anesthesiology, College of Medicine - Jacksonville, University of Florida, Jacksonville, USA; 2 Industrial Systems Engineering, Georgia Institute of Technology, Atlanta, USA

**Keywords:** bivariate, mortality, chloropleth, map, social determinants of health (sdoh), area deprivation index (adi), index of medical underservice (imu)

## Abstract

This investigation analyzed the impact of place-based inequities on mortality rates in 2014. The team combined mortality data with metrics on health care accessibility, socioeconomic deprivation, and other variables available from publicly available data sets. The investigation team created a centralized database for visualizations that combined mortality data by diagnosis, socioeconomic data, health resource data, and an index of area deprivation. Choropleth maps, scatterplots, and regression analyses were performed to identify the major areas of mortality and how well different measures of the social determinants of health (SDOH) correlate to mortality data. A bivariate color scheme to visually capture both outcomes and SDOH in a choropleth map was shown to be a compact and novel manner to display complex epidemiologic data.

## Introduction

According to the National Vital Statistics Reports from 2015 to 2017, for the first time in a century, the life expectancy of Americans either decreased or stayed flat [1]. Starting in the 1980s, rural regions have had higher mortality rates than urban areas. While there may be interdependence between rurality and education, the correlation between mortality and race or education appears to be weaker than rurality [2]. Due to the above-mentioned trends in life expectancy and the acceleration of increasing mortality in rural regions, an urgent investigation must be performed to better understand the inequities in health care, specifically for those in underserved areas [2].

This investigation analyzed the impact of place-based inequities on mortality rates in 2014. The team combined publicly available mortality data with metrics on health care accessibility and socioeconomic deprivation. With open-source data and software tools, an interactive dashboard was successfully created and made available at no cost for users to intuitively visualize and parse simultaneously the United States (US) mortality data and the social determinants of health (SDOH) at the county level. 

## Materials and methods

Mortality rates

Kochanek et al. completed significant work to understand mortality trends in more detail [1]. These reports assisted our model creation, exploration, and analysis by cause of death, race, and ethnicity. While the reports do provide differences at the state level, further granular analysis by county and the SDOH were not a focus of that report.

Determining underserved areas

Arcaya et al. outlined definitions and methodologies for studying health inequalities [2]. Among the most relevant concepts are the geographical concepts of place as a location within a governmental unit and space as a location without the concept of these boundaries. 

The Health Professional Shortage Areas (HPSA) and Medically Underserved Areas (MUA) are determined by Health Resource and Services Administration (HRSA) for incentivizing physicians to practice in underserved areas [3]. HRSA determines these areas via multiple criteria, including geographic area and population groups [4]. However, in Los Angeles, Juarez et al. found that currently identified HPSA covers less than a tenth of the two million people the authors identified as underserved via a physician to population ratio less than 1:3500 [5]. Assessing the impact of this discrepancy through health metrics, such as mortality, is a potential improvement.

Basu et al. found that a higher physician/population ratio reduced mortality rates [6]. However, this ratio is decreasing across the country. 

Other methods include average distance to a physician, the percentage of population further than a certain distance [7], travel time, hospital bed to population ratio, and an improved gravity method [8]. In addition, with new Application Programming Interfaces (API) and more powerful computers, some used the Geographic Information System (GIS) to determine block-level trip times [9]. Higgs categorized these different approaches, as shown in Table 1 [10].

**Table 1:** Definitions of Accessibility with Examples Adapted from Higgs [10]

Approach	Definition	Health Example
Container	The number of facilities contained within a given unit	Number of surgeries in a census ward
Coverage	The number of facilities within a given distance from a point of origin	The number of hospitals 10 km from a population centroid
Minimum distance	The average distance between a point of origin and the nearest facility	Distance between a village center and the nearest pharmacy
Travel cost	The average distance between a point of origin and all facilities	The average distance between the centroid of a census tract and all surgeries

Filling in gaps in data

To preserve anonymity, data sets, such as the Wide-ranging ONline Data for Epidemiologic Research (WONDER) from the Centers for Disease Control (CDC), censor out regions with a mortality count of fewer than 10 results [11]. As this has a detrimental impact on the understanding of health care issues in these regions, papers have studied how to fill in these gaps. Quick used Bayesian inferences to fill in missing data [11], and Liu and De used multiple imputations [12]. Woznicki et al. successfully used a random forest to map gaps that exist in floodplain data, which, while not healthcare-related, can still be leveraged [13]. 

Few of the above papers investigated correlations to mortality. Due to time constraints, the investigation will use HRSA and MUA data for accessibility and correlate these with mortality data. These findings will be compared against the work of Arcaya et al. to identify differences between the two accessibility approaches [2].

Stratification of the social determinants of health (SDOH)

While trying to understand healthcare accessibility holistically, Hawthorne et al. found that factors other than distance, such as poor listening skills of the doctor and poor quality of care, can drive people not to use local services [14-15]. Hawthorne et al. went on to use those findings to develop a satisfaction-adjusted distance metric that accounts for these additional variables from data generated from patient surveys.

**Figure 1:**
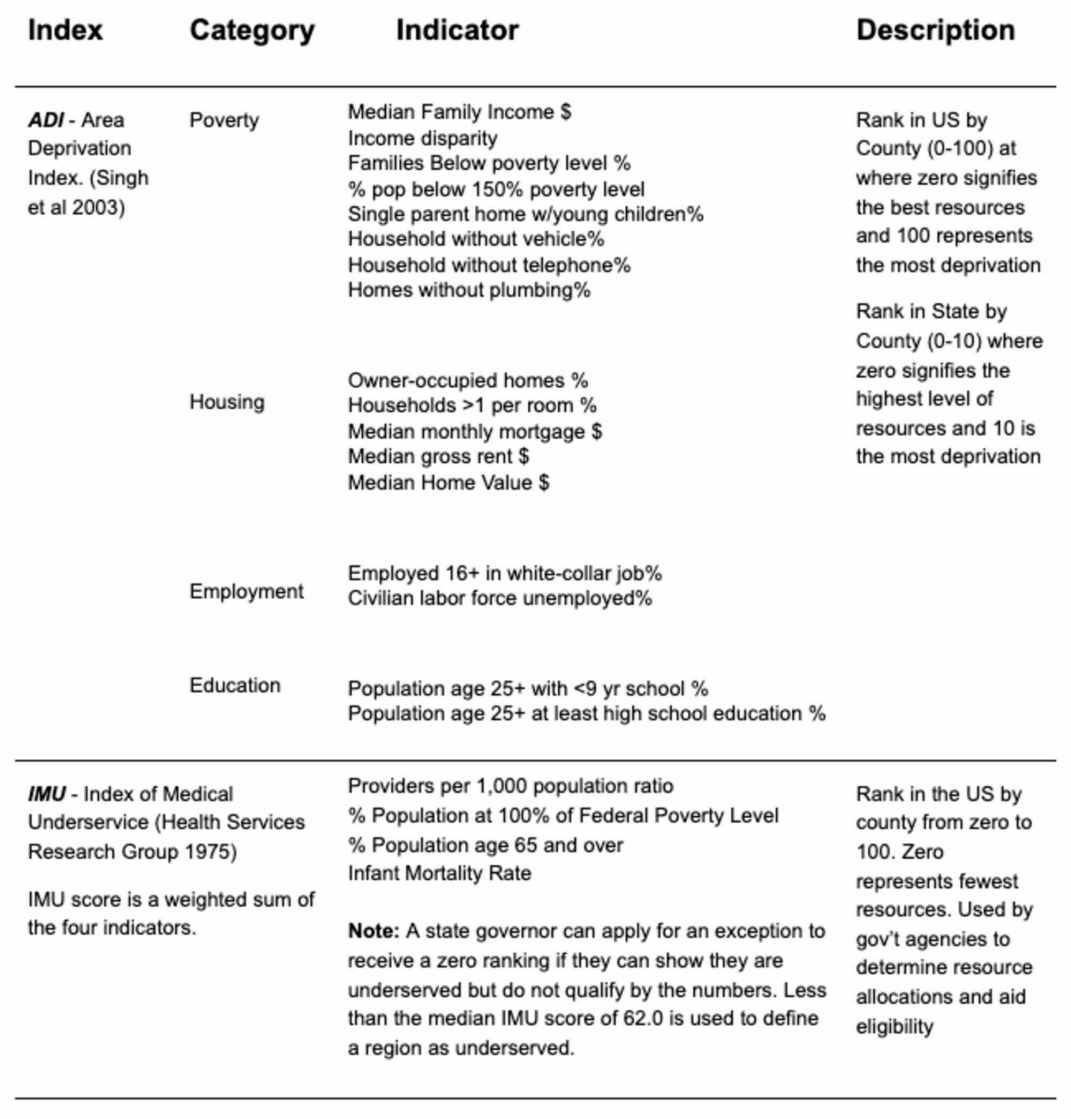
Social Determinants of Health (SDOH) - common health care indices and their components used to categorize regional healthcare resources and their availability into a composite ranking

**Figure 2:**
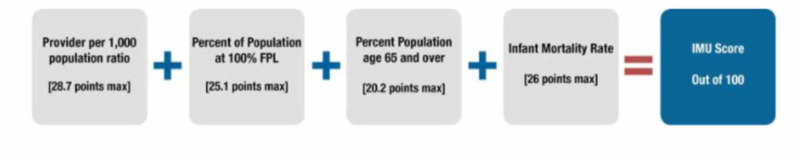
Health Resource and Services Administration (HRSA) calculation for the Index of Medical Underservice (IMU) score FPL: federal poverty level

Other methods include the use of binary, categorical, or ordinal data, such as sex, race, ethnicity, income, education, and healthcare needs, in their analyses [2]. Kind et al. developed the Area Deprivation Index (ADI) as a combination of factors that were shown to be a good indicator of readmissions in highly disadvantaged areas [16]. Regardless of the measure, there have been studies that have assessed a mortality penalty to poorer rural regions [17]. This investigation leveraged this metric (ADI), along with the Index of Medical Underservice (IMU), to analyze the HRSA and mortality data [18-19]. Figure 1 describes the factors that are used to derive the IMU and ADI, while Figure 2 demonstrates how the IMU is calculated. 

Visualizations

A commonly used visualization for health care accessibility is the choropleth map [2, 16]. Boscoe et al. identified that states, counties, and health service areas were ideal mapping units [4]. Data sets used in this investigation will be joined on the Federal Information Processing Standard (FIPS) county code. Brewer et al. identified that divergent schemes were better for identifying map clusters, and specifically, purple/green divergent schemes were the most pleasant [3]. These findings were utilized in maps generated in this investigation. 

Datasets, software, and hardware

The investigative team identified and retrieved data from the Global Health Data Exchange (GHDX), the socioeconomic data from the United States Census Bureau (USCB), HRSA’s MUA data, and the ADI. The data was collated and a common linkage identified: the FIPS code. FIPS is an identifier of counties across the US and served as the mapping unit on which analyses were based. Data sets for 2014 were the most recent where all sources were available. 

There are 3,142 counties in the US. At the county level, census and mortality data were procured for most of them. The process of cleaning data prior to database inclusion required several decisions: 1) several counties were modified to have a designation of partially rural as they had both rural and urban designations in the MUA database due to conflicting definitions amongst government agencies; 2) due to the irregular shape of counties and variability in health care factors and reporting at the US-Mexico international border, this factor was removed from the dataset; 3) the ADI provides data at the census tract and block level. As indicated in the study by Brewer et al., this level of detail is too fine-grained [3]. FIPS codes were parsed to provide county values and ranks averaged accordingly.

FIPS county codes were extracted from geographic identifiers (GEOIDs) to be able to link correctly with other data sets.

An SQLite database [20] was created from the above datasets with four main tables. The datasets were joined within Tableau to develop a series of dashboards to walk the user through various levels of analysis [21]. The choropleth maps on each dashboard were able to be filtered on the cause of death, region, state, and sex for further investigation. Per Boscoe et al., a green/purple divergent scheme was used where green indicates the lowest mortality/deprivation combination and purple the highest [4]. 

The first dashboard provides the user with an independent view of the two factors of interest, mortality, and deprivation, via a choropleth and histogram for each. Filters on poverty, unemployment rate, and minority population were added to filter choropleth counties [22].

The second dashboard contains graphs that enable the comparison of IMU and ADI. A bivariate choropleth map is provided for each mortality/IMU and mortality/ADI [22]. This type of map was selected for its ability to depict the contribution of each variable rather than combining the variables into a complex measure. This method of visualization was not found elsewhere by the investigation team during research and adds substantial insight. To create these maps, mortality, IMU score, and ADI national ranking were grouped into three buckets: high, medium, and low. The delineation for each set was one standard deviation on either side of the respective mean, taking all causes of death and both genders into consideration. Any null values were considered as low. Because an IMU score of 0 indicates the highest deprivation, the high/medium delineation is one standard deviation below the average, instead of above, as for mortality and ADI rank. 

When bucketing the data, counties with IMU scores of 0 were excluded as these are Governor’s Designated Shortage Areas and don’t receive scores via the standard calculation. The attached scatter plots also provide detail on the counties with the highest mortality and the top causes of death, thus allowing for comparison of the two indices.

The third dashboard is a divergent choropleth map displaying a complex measure to provide a quickly interpretable view of the trends across the country [22]. In the combined measure, the IMU score was multiplied by mortality to create a composite score. The IMU score was the selected deprivation index due to it being the most widely used. In addition, four charts with linked census data display more information on the populations included in the choropleth’s selected filters. Filters on poverty, unemployment rate, and minority population were added to be able to filter choropleth counties. 

The testbed environment was an operating system agnostic. Team members used Microsoft Windows (Microsoft® Corp., Redmond, WA, USA), Apple macOS (Apple, Inc., Cupertino, CA, USA), and Linux (Linux Foundation, San Francisco, CA, USA). Data was aggregated in SQLite [20]. The visualization application was Tableau Desktop 2019, Version 4 [21]. Data files were shared in an Office 365 OneDrive (Microsoft® Corp., Redmond, WA, USA) account set up through Georgia Tech. The final results and visualizations will be published through a Tableau public viewing website application.

Objectives of this investigation

The objectives of this investigation were to 1) visually identify trends between demographic data and mortality, 2) display the difference between the two major deprivation indices used in healthcare, 3) display correlation, if present, between deprivation and mortality, 4) improve the visual contrast for identification of the regions that may be over- or underserved, and 5) to create a compelling interactive visualization of the major causes of mortality at a local level, combined with the effect of being underserved, in order to rapidly target areas in a crisis that may be in need of further support.

## Results

Inspecting the demographic data shows that counties with high minority populations, high poverty rates, and high unemployment rates have higher average mortality rates. This corroborates the need for underserved indices that consider these factors. 

Instead of having to use multiple maps to display each concept, the team experimented with condensing the information into single choropleth maps using either multiplicative indexing (mortality multiplied by the IMU score) or bivariate color schemes (separating mortality and SDOH by standard deviations). 

**Figure 3:**
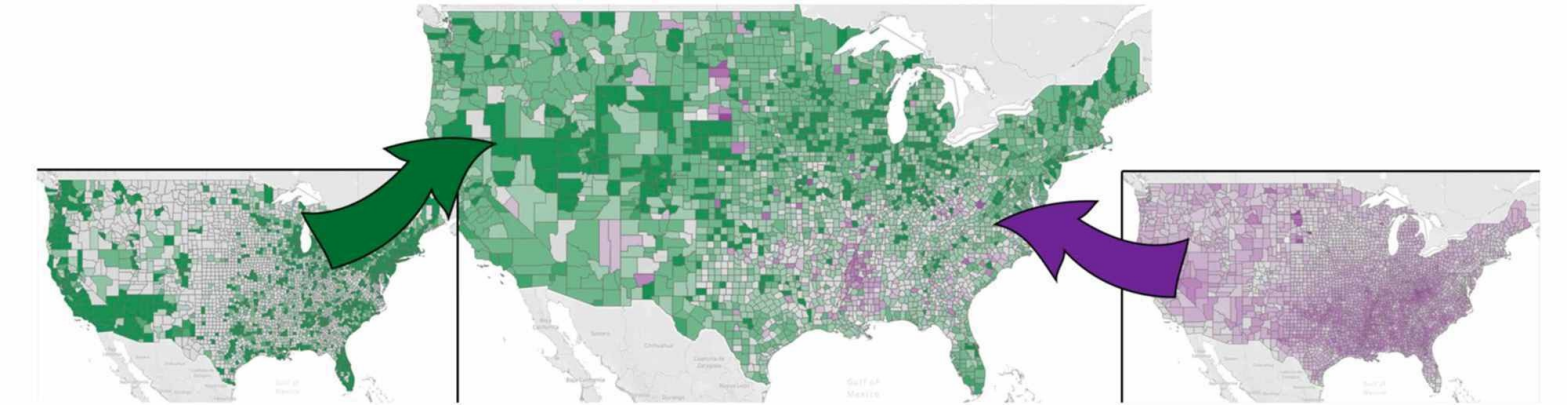
Choropleth Maps of Mortality/IMU Score Using a Multiplicative Index Mortality and Healthcare Access [22] IMU: Index of Medical Underservice

Figure 3 highlights the differences and allows for visualization of the relationships between mortality, ADI, and IMU. For all-cause mortality, when mortality/IMU is compared to mortality/ADI, it is visually apparent that the areas in the deep South and close to Native American reservations are better captured with the ADI in the bivariate color scheme. The scatter plots also exhibit more of a linear trend in the mortality/ADI plots.

**Figure 4:**
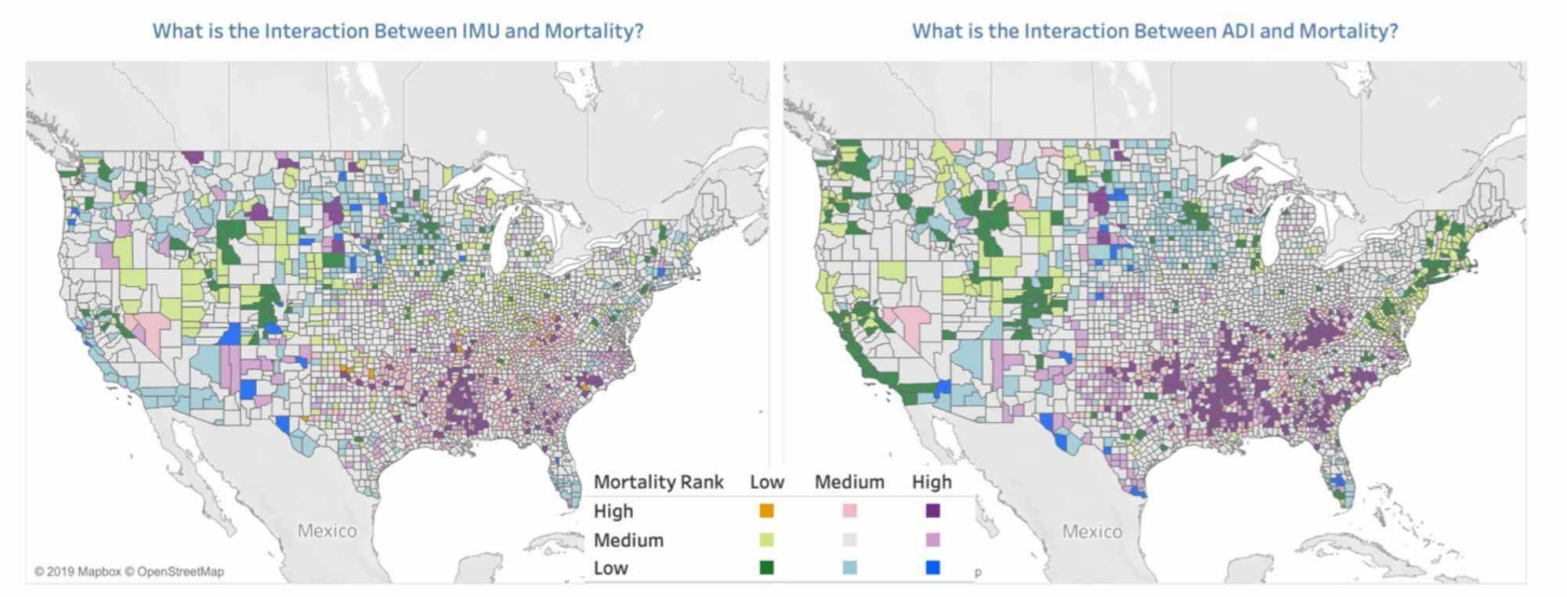
Bivariate Choropleth Maps of Mortality/IMU Score and Mortality ADI Rank Mortality and Healthcare Access [22] ADI: Area Deprivation Index; IMU: Index of Medical Underservice

**Figure 5:**
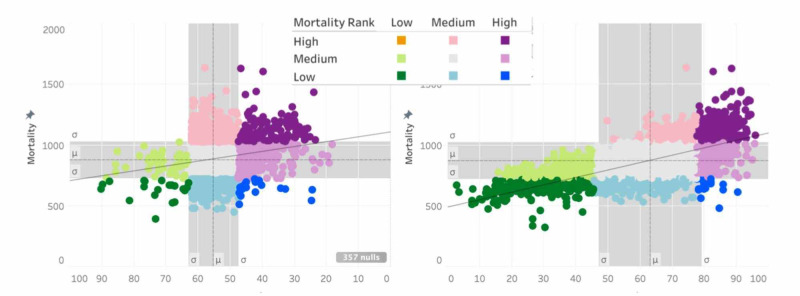
IMU vs ADI Scatter Plots with Fitted Regression Line Mortality and Healthcare Access [22] ADI: Area Deprivation Index; IMU: Index of Medical Underservice

Beyond the visual, regression analyses were performed on the two indices to compare correlation to mortality at the county level. Figures 4 and 5 graphically represent the best fit line for IMU and ADI versus all-cause mortality. While the p-value was less than 0.001 for each index, the R-squared coefficient was 0.41 for ADI and 0.04 for IMU. It is interesting that while IMU has been the standard for national governmental agencies in determining aid and assistance, the data supporting the relationship is barely better than random chance. This investigation concludes similarly to Liu and De that other methods for determining the underserved regions are stronger indicators of outcomes than HRSA’s IMU score [12]. The additional refinement of the IMU score built into the ADI greatly improves the correlation to mortality. 

From the top mortality counties, it is clear that ADI is more consistently categorized as high and a better representation of mortality than IMU score which varies between high and medium.

**Figure 6:**
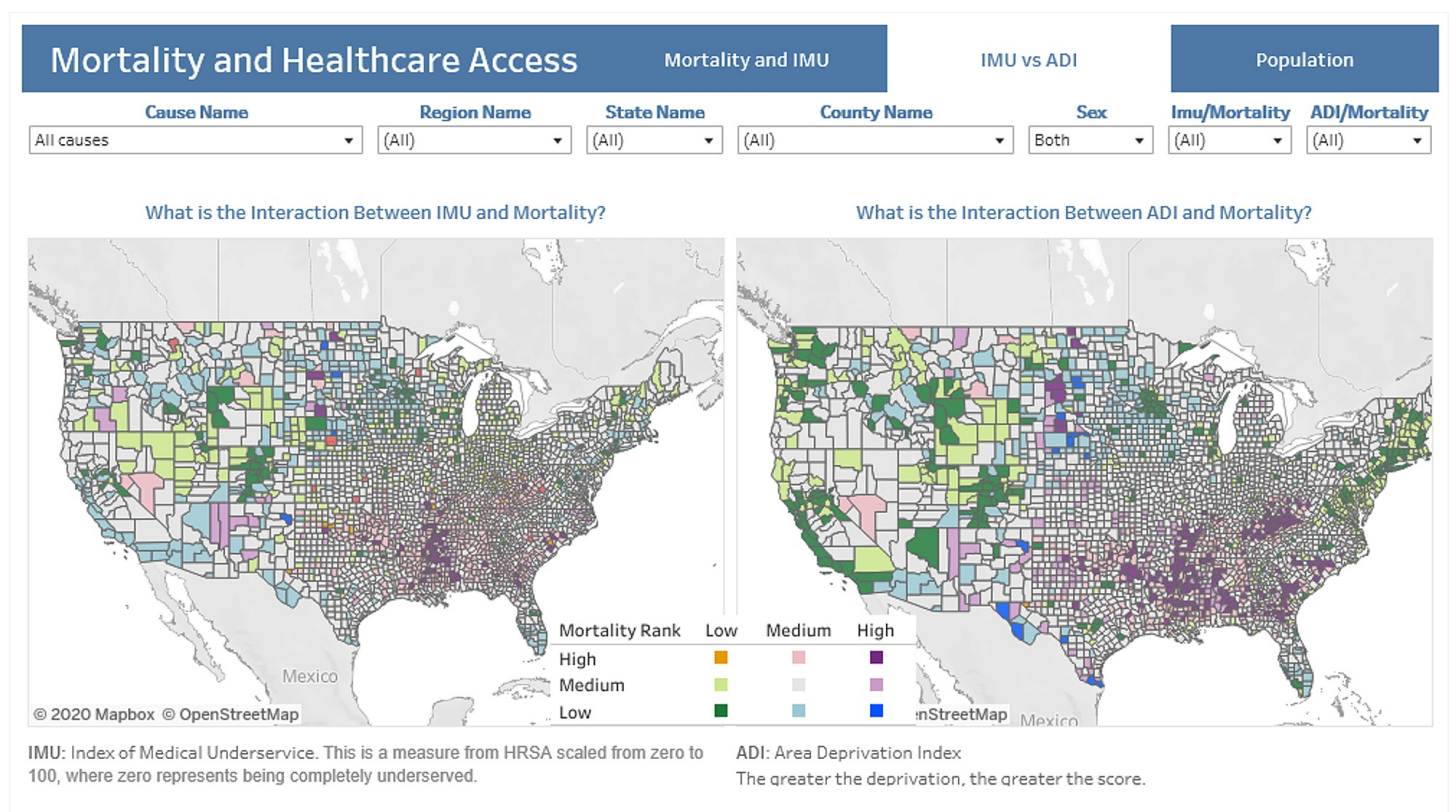
Dashboard Options and View for Underserved Indices Mortality and Healthcare Access [22]

For the leading causes of death, the largest category (noncommunicable disease) includes diseases not transmissible between people, such as heart disease, stroke, Parkinson’s, diabetes, and arthritis (Figure 6). Communicable diseases, such as human immunodeficiency virus (HIV), sepsis, hepatitis C, and sexually transmitted diseases, are smaller than their non-communicable cohort. Communicable diseases are often more prevalent in the underserved areas where people may not have the same access to education, prevention, and antibiotic therapy.

## Discussion

By visual inspection of the bivariate maps, a few concentrated pockets in the deep South and the Midwest near Native American reservations tend to be at the higher end of the indices and mortality groupings for all causes, cardiovascular disease, kidney disease, and neonatal mortality. These areas are more readily identified in the bivariate maps, especially when mortality is combined with the ADI rather than the IMU. Interestingly, at the county level, inner-city populations in affluent counties (New York, San Francisco, Los Angeles) seem to do better than inner-city populations in less affluent counties (Gary, Indiana; Mobile, Alabama; Akron, Ohio). This is a finding that should receive follow-up to see if this is accurate or if mortality data at the county level is masking problems that may be discoverable at the US census tract level. These observations lend further credence to the notion that there are spatial (urban, rural) and non-spatial factors (local economy, cultural values, and trust in medical institutions) that can affect outcomes [23].

An interactive version of the dashboard is available for interested users on a public Tableau server [22].

A number of innovations were developed as part of this investigation

Joining disparate datasets in a choropleth map that can be visualized at the county level for simultaneous health and socioeconomic data is a novel visualization undertaking. 

Using these datasets to create a bivariate visual model of mortality data, coupled with deprivation data, in a choropleth map is at the cutting edge of data visualization.

Linear regression models were created to compare the correlation of indices to mortality.

A template has been developed from both a visual and a database standpoint that could be easily built out in a modular fashion.

The multi-axis choropleth maps, when divided into groupings by standard deviation with drill-down capability, focus a spotlight on regions of medium and high underserviced/mortality that could be missed by focusing on single-axis or tabular data alone.

Future directions

1) Add past or future census and health resource data as it becomes available. This can be done intermittently or as part of a streaming database.

2) Add outcomes other than mortality. These can be health-related, but other possibilities include education, crime, or environmental outcomes. 

3) Add the CDC WONDER data set. This data set contains associations between mortality, demographics, and disease classification organized by the county FIPS code. It was not yet included as it would add approximately 300 million rows of data and would require imputation for missing or redacted data.

4) Further analysis of county mortality by diagnoses with models other than linear regression should be applied to determine if model accuracy can be improved.

5) Use models referred to in the immediate point above to generate visualizations and predictions about health and mortality by varying socioeconomic factors or index rankings. This would allow decision-makers to advocate for social and economic policies based on their predicted impact on health outcomes.

6) Release the dashboard to the public domain and perform split-run testing, also known as A/B testing, of various graphic features to determine which are getting the most traction from users. Combining multiple datasets into a compelling visualization with predictive capabilities has the added benefit of capturing users that may be searching for just one of the component datasets individually. 

7) Currently, the buckets used for the bivariate choropleth maps are fixed based on mortality due to all causes and do not adapt to other causes. Adding this feature would help with more detailed analyses.

8) The CDC, the Agency for Healthcare Research and Quality (AHRQ), and the Federal Emergency Management Agency (FEMA) provide links on their websites for open-source data on various outcomes by state and county [24-26]. Some concrete examples where this tool could be adapted include:

a) In 2019, the Agency for Healthcare Research and Quality (AHRQ) holds competitions to predict county-level data for different health care outcomes across the US based on only the previous data. The developed database and resulting models could help make more robust predictions and visualizations for such an effort.

b) The Federal Emergency Management Agency's (FEMA) damage claims data could be used in conjunction with a database and bivariate plots (like the one developed in this study) to examine the relationship between economic damages and health outcomes. That analysis could assist in anticipating resources required based on the path and severity of a future event.

c) A bivariate overlay of COVID-19 infection and outcomes combined with the SDOH to identify over and underperforming counties or census tracts. 

## Conclusions

During the investigation, some existing visualizations for individual characteristics were found. However, a gap was identified for visualizations that combined both US census and health care data into a multi-axis cohesive and visually appealing narrative. The investigation team was successful in creating an intuitive visualization that would allow a user to step through a multi-axis visualization at the national level down to individual diagnoses and socioeconomic factors at the county level. In addition, the team was able to make multiple charts and tooltips that vary with the selection of a region of interest. 

The team focused on the association of deprivation indices and their relationships to mortality. While this relationship is not necessarily causative, it is the team’s belief that improving or worsening these social determinants will affect outcomes in either a direct or indirect manner. It was found that the commonly used IMU score does not have a strong correlation to mortality, whereas ADI has a stronger correlation. As society changes and new technologies evolve, there will be a need to revise the indicators of the undeserved to better reflect reality. Models and visualizations that capture these associations will provide insights to better identify and track changes in an effort to better support underserved areas and improve mortality rates.
